# Supplementary material for: Are carbon emissions trading and green financial instruments synergistic? -Comprehensive quantitative research based on content analysis
Source: PLoS One. 2024 Mar 7;19(3):e0298601. doi: 10.1371/journal.pone.0298601 (PMC10919601; doi:10.1371/journal.pone.0298601)
Supplement: S1 Data — (ZIP) [file pone.0298601.s001.zip › Data/地级市Z_F工作报告.zip!/║╙─╧/╓ú╓▌╩╨/└┤╫╘╣1⁄2╓┌║┼í╛┬φ┐╦╩2╛▌═°í┐_2014.docx]

各位代表：

　　现在，我代表市人民政府向大会报告工作，请予审议，并请各位政协委员提出意见。

　　一、过去五年的工作回顾

　　过去五年，是郑州发展进程中很不平凡的五年。面对错综复杂的经济形势和激烈竞争的发展环境，我们在省委、省政府和市委的正确领导下，坚持以邓小平理论、“三个代表”重要思想、科学发展观为指导，高举中国特色社会主义伟大旗帜，紧紧依靠全市人民，一心一意谋发展，凝心聚力促转型，抢抓机遇求突破，完善机制抓落实，全面完成了市十三届人大一次会议确定的目标任务。

　　(一)着力促进经济社会持续健康快速发展，综合实力再上新台阶。五年来，面对国际金融危机的持续冲击和经济形势复杂多变的严峻挑战，我们扭住发展不放松，圆满完成“十一五”目标，顺利实施“十二五”规划。特别是近两年来，面对较大的经济下行压力，我们抢抓中原经济区和郑州航空港经济综合实验区上升为国家战略的重大历史机遇，突出“三大主体”工作，坚持开放创新双驱动，着力“保增长、调结构、促转型、惠民生”，加快推进郑州都市区建设，经济社会呈现持续健康快速发展的良好态势。2013年，全市地区生产总值完成6202亿元，年均增长12.1%，是2008年的1.8倍；地方财政总收入1116亿元，年均增长19.1%，是2008年的2.4倍；公共财政预算收入723.6亿元，年均增长22.7%，是2008年的2.8倍；固定资产投资4400亿元，年均增长23.8%，是2008年的2.9倍；进出口总额422亿美元，年均增长76.1%，是2008年的12.3倍，占全省总量70%以上；社会消费品零售总额2586亿元，年均增长15.8%；城镇居民人均可支配收入26615元，农民人均纯收入14009元，年均分别增长11.1%和13.2%，城乡居民收入比由2.1︰1缩小到1.9︰1。经济发展质量和效益明显提升，科技进步贡献率达到58%，单位生产总值能耗五年下降20.3%。先后被评为全国科技进步示范城市、国家创新型试点城市和全国文明城市。

　　(二)积极转变发展方式，结构调整迈出新步伐。大力实施“工业强市”战略，现代工业体系加快构建。五年累计完成工业项目投入5788.5亿元。2013年，规模以上工业增加值完成2857.7亿元，年均增长15.9%，占全市生产总值比重46%；着力打造“6+2”千亿级产业基地，工业七大主导产业销售收入达到8000亿元，占全市规模工业的比重达到67.6%，对工业增长贡献率达到80.4%，其中汽车及装备制造业销售收入达到2100亿元，电子信息产业销售收入超过2000亿元，新材料产业销售收入突破1500亿元。突出服务业优先发展，现代服务业提质增效。2013年服务业固定资产投资完成2964亿元，年均增长26.9%；产业增加值完成2584亿元，年均增长10.2%；全市新增金融机构69家，郑东新区金融业集聚效应初现；会展业快速健康发展，服务业综合改革、旅游综合改革试点城市建设和现代物流、电子商务示范城市建设扎实推进。大力发展现代都市农业，农业农村基础地位进一步巩固。全市粮食总产量稳定在165万吨，连续十年喜获丰收。“菜篮子”保障能力持续增强，现代都市农业示范区建设“136”工程成效明显，农业产业化、科技化水平稳步提升，市级以上农业产业化龙头企业达425家，加工型农业龙头企业销售收入突破500亿元。农产品质量安全监管体系不断完善，检测合格率位居全国前列。坚持“四集一转”，产业集聚区承载能力明显提升。2013年产业集聚区主导产业完成营业收入4600亿元，占集聚区全部营业收入的60%；航空港产业集聚区成为全省首个超千亿产业集聚区，高新区、经开区产业集聚集群水平明显提升；2个商务中心区和11个特色商业区规划建设全面启动。加快自主创新体系建设，科技创新能力进一步提高。2013年，市级以上研发中心1705家；高新技术企业346家，比2008年增加225家；专利授权量1.2万件，是2008年的3倍；国家技术转移郑州中心成功落地，国家专利审协河南中心建成运行；国家专利导航产业发展实验区试点工作有序推进，国家质检中心郑州综合检测基地开工建设；通过第二批国家级两化融合试验区验收，获评国家知识产权示范城市、下一代互联网和全国数字城市建设示范城市，成为电子商务、信息消费、智慧城市试点和国家互联网骨干直联点城市。加大节能减排力度，淘汰落后产能。关闭煤矿51家，淘汰落后煤炭产能729万吨，取缔污染严重企业412家，实施重点节能减排项目186个，资源型产业比重降低到45.8%，比2008年降低11个百分点。

　　(三)加快推进城乡一体化发展，城乡面貌发生新变化。全面实施新一轮城市总体规划。先后编制并获批郑汴新区空间发展战略规划、郑州都市区总体规划、航空港实验区总体规划等重要规划，现代城镇体系架构初步建立。加快都市区交通路网体系建设。域内畅通工程加快实施，“井字+环线”快速路网加快建设，京广快速路一期、三环快速化主线工程建成投用，陇海快速路、环城高速出入市口等工程快速推进，地铁1号线一期如期试运营，10条市域快速通道基本建成，打通城区断头路110条，综合整治道路237条，改造积水点53个，新增停车位14万个，新改建县域道路3408公里；域外枢纽工程扎实推进，郑西、石武高铁全线通车，郑徐高铁、郑开、郑焦及郑州至机场等城际铁路加快建设，郑州东站、火车站西广场建成投用，西绕城、郑云、郑民、巩登高速建成通车，机场二期工程快速推进，综合交通枢纽地位明显提升。着力提升中心城区和县城组团现代化建设水平。郑东新区顺利实现“十年建新城”预定目标；中心城区建成区面积由303平方公里扩大到346平方公里；全面启动生态廊道、县城十字景观大道建设，13个组团新区新增建成区27平方公里；三环以内完成87个整村拆迁和76个市场外迁；城镇化率达到67%。扎实推进“四类社区”建设。实施社区建设项目449个，开工安置房项目427个，回迁居民33.6万人。持续完善城市基础设施建设。一大批水、电、气、暖和污水、垃圾处理等市政工程建成投用，城市污水处理率、垃圾无害化处理率分别达到85%、93%，城市集中式饮用水源地水质达标率达到98%。努力改善城乡环境。启动国家森林城市和生态园林城市创建工作，完成植树造林95万亩，新增城市绿地4103万平方米，成功举办第二届全国绿博会，郑州绿博园、植物园等一批大型公园建成开放，惠济区成为我市首个国家级生态示范区；实施都市区生态水系建设，龙湖调蓄工程建成蓄水，我市被确定为全国水生态文明试点城市；南水北调中线郑州段提前完工，配套工程扎实推进；实施“蓝天”工程，开展PM2.5监测，加强重点污染源整治，拆改市区燃煤锅炉656台，淘汰黄标车5200辆，完成2家热源厂煤改气工程；实施城市精细化管理，城区环境整治成效明显。

　　(四)深化体制机制创新，改革开放实现新突破。大力推进重点领域和关键环节改革。推行城乡规划土地建设管理“六统一”改革，提高政府调控能力和资源配置效率；推进各类组团新区、产业集聚区管理机构与行政区域管理套合制度改革，构建了“小政府、大服务”的管理体制；全面推进行政审批制度改革，审批事项由363项削减至141项，削减64.2%，审批环节压缩61.2%，总承诺办结时限减少79.5%；深化投融资体制改革，整合组建政府八大投融资公司，初步建立了立项、融资、使用、管理、偿债“五位一体”的全产业链发展模式；深化金融体制改革，郑州银行、百瑞信托完成股份制改造，新建村镇银行6家；推进企业战略重组和改制上市，全市上市公司达37家，12家科技型中小企业成功登陆“新三板”；优化民营企业和中小企业发展环境，非公有制经济快速发展，占全市生产总值比重达61.1%；深化税制改革，稳步推进“营改增”试点工作；国有企业、事业单位等改革取得新进展；顺利完成“五险合一”市级统筹工作，社会保险管理服务水平全面提升。持续加强开放平台及招商机制建设。推动郑州航空港经济综合实验区上升为国家战略，全面启动郑州通航实验区建设，全力打造对外开放战略高地，航空港实验区建设实现良好开局；新郑综合保税区封关运行，郑欧班列顺利开通，肉类、药品进口口岸获批筹建，跨境贸易电子商务服务试点实施，电子口岸和国际陆港建设加快推进，明显提升了我市对外开放水平；建立招商引资工作新机制，承接产业转移取得显著成效，2013年实际到位域外资金1497亿元、是2008年的3.4倍，实际利用外资33.2亿美元、是2008年的2.4倍，来郑投资或设立机构的世界500强企业达83家、比2008年增加64家。

　　(五)着力保障和改善民生，各项社会事业取得新进步。始终坚持发展为了人民，切实让人民群众共享改革发展成果。持续加大民生投入，全市财政用于民生领域的支出五年累计达1856.9亿元，年均增长26.8%，省、市确定的民生“十大实事”全部完成。努力扩大就业，新增城镇就业人员69.2万人，城镇登记失业率控制在4%以内；发放小额担保贷款61.1亿元，拉动就业21.2万人；培训农村劳动力36.6万人，转移就业59.1万人。提高社会保障水平，社会保障实现制度全覆盖，标准和待遇逐年提高，企业退休人员人均养老金从每月1097元提高到1992元，城镇居民基本医保基金支付比例提高5个百分点，新型农村合作医疗人均筹资标准从90元提高到340元，城乡低保标准分别提高155元和130元，达到每人每月430元和240元；新建、改扩建敬老院38所，新建社会养老机构27家，新增床位6100张；积极推行保障性住房“三房合一”，开工建设各类保障房21万套，60余万人纳入住房保障范围；改造老旧小区1968个，受益群众28万户。优化教育资源配置，新建、改扩建幼儿园305所、市区中小学校143所，新增中小学学位15.8万个、幼儿园学位8.6万个；郑州师范学院成功升本，中州大学、旅游职业学院、职业技术学院等高校提升发展。文化惠民工程稳步实施，成功创建全国公共文化服务体系示范区，群众性文化活动不断丰富，“天地之中”历史建筑群成功申遗，拜祖大典、少林武术节、嵩山论坛等品牌效应不断扩大，舞剧《水月洛神》囊括四项国家级大奖，豫剧《斗笠县令》获中国戏剧节优秀剧目奖。全民健身运动蓬勃发展，竞技体育水平不断提高，我市运动员在伦敦奥运会上勇夺2枚金牌。医疗卫生服务水平持续提升，在全国率先实施“片医负责制”社区卫生服务模式，新农合经办机制改革“郑州模式”在全国推广，一批医疗卫生基础设施建成投用，扩增医疗床位2.8万张。农村生产生活条件持续改善，改造中低产田80万亩，解决300万农村人口饮水安全，完成易地扶贫搬迁1.5万户。群众出行环境不断优化，绕城高速免费通行，二级公路收费站全部撤销，普通干线公路回归公益；新增更新公交车3128台，新建公交场站、公交港湾235个，入选全国“公交都市”建设示范工程首批创建城市。慈善事业快速发展；社会救助帮扶体系进一步完善，市救助站被授予国家一级救助管理机构；低生育水平持续稳定，成为全国人口和计划生育综合改革示范市、全国流动人口均等化服务综合改革试点城市。

　　(六)加快政府职能转变，社会治理能力得到新提升。自觉接受人大、政协和社会各界监督，办理议案建议提案5492件，制定政府规章33件，办理行政复议案件3985件，向市人大提交地方性法规草案13件。健全重大决策公众参与、专家咨询论证评估等制度，完善政务和信息公开。审计监督、行政监察和行政层级监督不断强化。大力推进节约型机关建设，在“三公”经费保持零增长的基础上，2013年比2012年压减11.6%。建立健全以网格为载体坚持依靠群众推进工作落实长效机制，构建了“三级网格、四级平台、五级联动”的工作架构，形成了以党组织为核心，政府市场监管、社会管理和公共服务与群众自治有效衔接、互为支撑的基层治理结构，基层网格排查问题化解率保持在97%以上。创新社会治理机制，社区建设管理服务工作明显加强，城乡社区和基层自治组织服务功能进一步健全。加强法制宣传教育。扎实开展平安郑州建设，深入推进“四严一创”等专项行动，获评全国社会管理综合治理优秀市。强化安全生产、消防安全和食品药品安全监管，社会大局保持和谐稳定。

　　加强社会主义核心价值观教育，社会文明程度不断提高。国防教育和国防后备力量建设取得新成效，“中华神盾”郑州舰入列东海舰队，创建全国双拥模范城实现“六连冠”。圆满完成四川江油灾后援建、新疆哈密对口支援和南水北调丹江口库区移民搬迁安置任务。外事、侨务、对台工作不断加强，民族团结、宗教和谐局面持续巩固，统计工作服务科学决策、反映经济社会发展的能力明显增强，社会科学繁荣发展，档案管理和史志编纂质量逐年提高，防震减灾、气象服务和人防应急准备能力进一步提升。各位代表！成绩令人鼓舞，经验弥足珍贵。五年来，我们始终坚持科学发展。紧紧结合郑州实际，全面贯彻落实科学发展观，坚持走好以“一个载体、三个体系”为支撑的“三化协调、四化同步”科学发展路子。始终坚持加快发展方式转变。紧紧围绕建设资源节约型和环境友好型社会，着力促进结构调整和经济转型，加快构建现代产业体系，不断提高城市核心竞争力。始终坚持统筹城乡发展。按照“统筹发展、协调发展、均衡发展”理念,制定完善提升郑州都市区建设规划，努力形成合理的城镇体系、合理的人口布局、合理的产业布局和合理的就业结构，促进基本公共服务均等化，加快推进城乡一体化。始终坚持关注民生。不断强化拓宽服务民生的理念和路子，着力在保障和改善上做文章，着力在服务质量和服务方式上求提升，以改善民生凝聚民心，以凝聚民心汇聚民力，以汇聚民力共促发展。始终坚持改革创新。突出问题导向、市场导向、需求导向，强力推进重点领域和关键环节改革，不断推动体制机制创新，持续增强发展的内生动力和活力。始终坚持党委领导。在党委统一领导下，全面履行政府职能,统筹各方力量，建立健全责任、有序、高效的工作推进机制，形成推动改革发展的强大合力。我们相信，只要我们坚持这些好思路、好机制、好举措不动摇，只要我们坚持一张蓝图绘到底、一届接着一届干，郑州都市区建设的美好愿景就一定能够实现！

　　各位代表！五年来，在外部经济环境持续影响、我市结构性矛盾凸显的情况下，取得这些成绩实属不易。虽然2013年的经济增速和部分指标低于年度预期，但仍处高速增长区间，不仅高于全国全省平均水平，而且结构、质量、效益等指标明显好于预期。这是省委、省政府和市委正确领导的结果，是市人大、市政协和社会各界大力支持的结果，是全市人民团结奋斗的结果。在此，我代表市人民政府，向辛勤工作在各行各业的广大干部群众，向驻郑解放军、武警官兵、公安民警以及社会各界人士，向关心支持郑州发展的港澳台同胞、海外侨胞和朋友们，表示崇高的敬意和衷心的感谢!

　　成绩面前，我们也清醒地认识到存在的问题和不足，主要表现在：生态环境污染尚未得到有效治理，雾霾等环境问题比较严重；城市基础设施建设滞后于城市发展，交通拥堵问题比较突出；科技创新能力不足，产业结构不尽合理，高新技术产业规模小，第三产业比重不高、现代服务业发展滞后；城乡二元结构和城市内部二元结构尚未得到有效改善，城镇居民和农民持续增收难度不断加大；就医、就学、就业条件与群众需求还有不小差距，安全生产、食品药品监管等还存在着薄弱环节；政府职能和作风转变还不到位，个别部门和工作人员依法行政观念和为民服务意识淡薄，行政效能和公共服务水平还需要进一步提高等。对于这些问题，我们将在今后工作中采取有效措施，尽快加以改进和解决。

　　二、今后五年的奋斗目标

　　今后五年，是我市紧紧围绕“三大一中”战略定位，全面完成“十二五”目标、谋划实施“十三五”规划的关键时期，是在全省率先全面建成小康社会的决定性阶段。我们既处于“爬坡过坎、攻坚转型”期，更处于“抢抓机遇、奠定基础、确立地位”的关键期。面对新形势、新任务、新要求，我们一定要紧紧抓住中原经济区和航空港实验区建设两大历史机遇，扭住结构调整、经济转型、产业升级不放松，全力打好郑州转型发展、跨越发展这场硬仗，着力奠定综合交通枢纽基础、现代产业基础、可持续发展的生态基础和良好的政务环境基础，初步建成自然之美、社会公正、城乡和谐的现代化都市区，初步确立以国际化、现代化立体综合交通枢纽为特征的国家中心城市地位。

　　今后五年政府工作的总体要求是：坚持以邓小平理论、“三个代表”重要思想、科学发展观为指导，认真贯彻落实党的十八大和十八届二中、三中全会精神以及中央、省委和市委的各项决策部署，牢牢把握主题主线，按照“三大一中”战略定位，围绕“一个载体、三个体系”建设，抢抓改革发展机遇，突出“三大主体”工作，坚持开放创新双驱动，着力提高经济发展的质量和效益，着力保障和改善民生，加快推进以航空港经济综合实验区为统揽的郑州都市区建设，全面推进经济、政治、社会、文化、生态文明建设，努力到2018年在全省率先全面建成小康社会，为建设“四个河南”、推进“两项建设”做出省会城市应有的贡献。今后五年，努力实现以下主要目标：

　　——初步形成具有较强竞争优势的现代产业体系。综合经济实力显著增强，经济结构战略性调整取得明显成效。2018年全市地区生产总值突破1万亿元，人均地区生产总值突破10万元，地方公共财政预算收入突破1000亿元，大都市战略支撑产业体系基本确立。汽车及装备制造业核心竞争力凸显，建成全球最大的客车生产基地、新能源汽车研发和生产基地，年产汽车整车超过150万辆；电子信息产业集群发展，成为全球最大的智能终端基地、国内领先的电子信息产业基地；现代商贸物流业规模进一步壮大，以郑欧班列多元化运营和郑州国际陆港建设为支撑，全力打造中欧铁路运输中心节点城市和新丝绸之路经济带“桥头堡”，依托上街机场加快发展通用航空产业；着力构建集约化、专业化、组织化、社会化有机结合的新型农业产业化经营体系，全市农产品加工型龙头企业销售收入达到800亿元以上；文化创意旅游业快速发展，产业增加值及旅游总收入均突破1000亿元；高新技术产业占规模以上工业比重达到30%以上，研究与开发经费支出占生产总值比重达到3.5%以上。

　　——初步形成城乡一体化发展格局。深入推进城乡统筹发展，加快实现城乡共同繁荣。中心城区功能完善，卫星城与中心城市协调发展，产业集聚区和专业园区融城发展，城市现代化水平明显提升，城镇化率达73.5%以上。城市基础设施和公共服务设施向农村延伸，城乡公共设施共享水平不断提高。新型农村社区建设取得明显成效，建成一批各具特色的“美丽乡村”。城乡居民收入大幅提高，中等收入群体持续扩大，人民群众过上殷实富裕的生活。基本公共服务均等化初步实现，全民受教育程度和创新人才培养水平明显提高，教育现代化基本实现；人人享有基本就业创业机会，人人享有基本社会保障，人人享有基本医疗卫生服务，公共文化服务体系和住房保障体系基本建立；每万人拥有医疗床位达70张以上，每千名老人拥有养老床位33张，人均体育场地面积达1.8平方米。

　　——初步形成国际化、现代化立体综合交通枢纽。构建连接世界的空中通道，新郑机场新开通国内外货运航线30条、客运航线50条，航空货邮吞吐量达到110万吨以上，客运吞吐量达到2300万人次以上，成为重要的航空枢纽；加快推进上街机场改扩建工程，加强产业布局和功能完善，争取建成中原经济区通用航空枢纽机场；“米”字形快速铁路网络骨架基本形成，郑州南站随郑万高铁建成运营，形成以郑州为中心、辐射省辖市的“1小时交通圈”和周边省会城市的“2小时交通圈”；以郑州为中心的“十字+半圆”城际铁路网加快建设，基本形成中原城市群核心区“半小时交通圈”；地铁轨道交通力争通车里程达130公里以上，“三横两纵一环”轨道线路覆盖城区；“畅通郑州”工程完成“井字+环线”快速路网建设和“两环三十一放射”改造提升，三环以内断头路全部打通，主城区实现15分钟上快速路、沿快速路15分钟上高速的“市区半小时交通圈”。

　　——初步形成重要的内陆开放高地。以航空港实验区为主平台，以发展航空货运为突破口，加快郑州国际空港、国际陆港建设，完成机场二期工程、新郑综保区三期工程和经开综保区申建工作，力争郑州自由贸易园区申建获批，航空港开放门户地位基本确立。郑欧班列、跨境贸易电子商务规模化运营，食品、药品、汽车整车等特种商品进口指定口岸功能不断完善，完成电子口岸平台建设，通关更加便利化。对内开放和对外开放并举共进，内外资、内外贸一体化运作，引进来与走出去融合发展，引资、引技、引智有机结合，集聚一批具有国际竞争力的知名品牌和优势企业，全方位、宽领域、多层次、内外融通、流通便捷的开放格局更加完善，开放型经济的带动力和贡献度显著提升。

　　——初步形成可持续发展的生态环境基础。以创建国家生态文明先行示范区为统揽，加强环境保护，加快生态建设，为人民群众创造良好的生产生活环境。开展国家森林城市和国家生态园林城市创建工作，实施林业生态市建设提升工程，城市建成区绿地率达到36.5%以上，绿化覆盖率达到42%以上，人均公园绿地面积11.5平方米以上，森林覆盖率达到34%以上。建设全国水生态文明试点城市，提升都市区生态水系规划建设水平，形成“一环、三源、六区、十八点”的生态水系总体格局，规划区水面面积达到115平方公里。着力改善大气环境质量，加快淘汰落后产能，推进清洁能源替代利用，大幅削减污染物排放量。强力推进“引热入郑”工程，开辟裕中热电、荥阳国电等外围热源，确保2015年完工投用，市区新增供热面积2500万平方米；强力推进“热电外迁”工程，今年实质性启动，力争2016年底实现新力电厂5台20万千瓦燃煤机组从市区迁出；强力推进“煤改气”工程，加大市区现存156台燃煤锅炉拆改力度，确保2015年拆改任务大头落地，基本达到“直排为零”。完成上述三大工程，可累计新增供热面积3100万平方米，减少中心城区用煤839.5万吨，减排烟尘6817吨、二氧化硫2.7万吨、氮氧化物4.9万吨，2018年PM2.5平均浓度比2013年下降20%以上！

　　——初步形成公开、公平、公正的政务环境。以保障权利公平、机会公平、规则公平为核心，着力打造行为规范、运转协调、公正透明、廉洁高效的政务环境。健全政务公开制度，深化行政审批制度改革，强化网上审批、行政电子监察系统建设，增强政府工作透明度；加强行政效能建设，强化服务意识，改进管理方式，规范行政行为，提高工作效率和水平；坚持依法行政，严格行政执法监督，完善执法责任制和执法过错追究制；加快推进信用体系建设，营造诚实守信的社会环境；正确处理政府和市场的关系，使市场在资源配置中起决定性作用，资源配置效率和公平性明显提高。

　　各位代表！在全省率先全面建成小康社会，是人民群众的殷切期盼，是历史赋予的光荣使命！我们坚信，经过五年的不懈努力，一个更有实力、更具活力、更富魅力的郑州都市区必将展现在世人面前！

　　三、2014年的目标任务

　　今年经济社会发展的主要预期目标是：全年地区生产总值增长10%以上；规模以上工业增加值增长12%以上；固定资产投资增长23%以上；地方财政公共预算收入增长14%以上；社会消费品零售总额增长13%以上；进出口总额增长6%；实际利用外资增长5%；城镇居民人均可支配收入实际增长8%以上；农民人均纯收入实际增长8%以上；城镇新增就业13万人；居民消费价格指数控制在3.5%左右；节能减排完成省定目标。

　　我们重点做好以下工作：

　　(一)全面推进航空港经济综合实验区建设，着力打造中原经济区核心增长极。围绕“打造大枢纽、发展大物流、培育大产业、形成大都市”，以完善基础设施和招商项目落地为重点，力争地区生产总值增长30%以上，固定资产投资翻一番。加强基础设施建设。全力推进新郑机场二期及配套工程、与主城区连接的“四纵六横”快速路网、城际铁路及区域交通路网、水电气暖厂站、生态水系、学校、医院等基础设施建设，增强货物集疏和项目落地支撑能力。优化国际营商环境。加快综保区三期、河南电子口岸平台等项目建设；推进跨境贸易电子商务试点规模化运营，初步形成国际网购品集散分拨中心；推进汽车整车进口口岸申报，加快药品、肉类进口口岸建设；推动国家重点通讯检测实验室和食品、药品、医疗器械检验检测中心建设。加快高端产业集聚发展。抓好航空设备制造维修、电子信息、生物医药重大项目集群式招商落地，重点推动菜鸟(中国)智能骨干网、手机产业园等建设，力争全年签约项目45个以上、开工30个以上。提升港区城市功能。以南区高端制造业集聚区为重点，完善市政配套设施，加快航空都市建设，构建绿色宜居生活环境和集约有序城市空间。推动郑州通航实验区加快建设。支持上街机场改造升级，完善基础设施建设，做好产业项目招商，促进通用航空产业发展。

　　(二)加快经济结构调整，着力构建现代产业体系。强化工业主导地位。实施工业主导产业提升规划，着力推进重点产业集聚、集群发展。打造以电动汽车为引领的新能源产业和汽车产业集群，力争汽车及装备制造业年销售收入达到2500亿元；打造以智能手机为代表的智能终端产业集群，力争电子信息产业年销售收入达到2800亿元；打造物联网、北斗导航等新兴产业集群，力争新材料、生物医药等战略新兴产业年销售收入突破2000亿元；实施战略性企业(集团)培育行动计划，力争全市年销售收入超百亿元企业达到12家；促进东风日产30万台整车项目等151个重点签约项目早开工，力争河南中烟易地技改等108个项目早达产；提升传统产业“两化”融合水平，加强工业质量品牌建设，全力化解过剩产能。促进服务业提质增速。加快建设全国重要的现代服务业基地，力争全市服务业增加值达到2900亿元以上、固定资产投资完成3700亿元以上；大力发展以电子商务为引领、以营造平台为核心的生产性服务业，以中心商务功能区和特色商业区为带动的生活性服务业；加快航空物流、铁路货运和公路物流基础设施建设，推进国际物流园区等物流项目建设；推进都市区商业中心建设，加快市场外迁，完成外迁市场54个；积极引进各类金融机构，加快金融集聚核心功能区发展；培育优势文化创意旅游产业，建设旅游集散中心和智慧旅游城市；大力发展高技术服务业，规划建设国家级会展中心，鼓励支持发展养老产业，积极推进国家服务业综合改革试点工作。加快发展现代都市农业。抓好高标准农田建设、“菜篮子”工程、现代都市农业示范区“136”工程、农业产业化集群培育四大工程，建成高标准农田20万亩，开发新菜田4万亩，新增设施农业5000亩，农业产业化集群达到20个；大力发展都市生态农业和休闲观光农业，发展新型农业经营主体，建设农产品质量安全追溯体系，建立现代农业产业技术体系。加快重大项目建设。力争今年列入省重点项目数量和总投资规模占全省比重均达到50%以上，新开工省、市重点项目240个以上，竣工90个以上，完成投资3300亿元以上。发挥政府性资金引导作用。集中使用产业发展专项资金，重点支持主导产业发展和重大项目建设；探索设立产业发展引导基金，建立以财政资金为引导、社会资金为主导的产业投资体系。

　　(三)坚持以人为本，着力提升新型城镇化发展质量。以人的城镇化为核心，提高新型城镇化的科学性和实效性。坚持规划引领。持续优化全域空间布局规划，提高规划水平和建设标准；严格林地、耕地、园地保护，形成边界红线，促进人口增长与产业发展、基础设施和生态环境承载能力相协调。有序推进农业转移人口市民化。加快户籍制度改革，全面放开建制镇和县城落户限制，推动城中村、城郊村和产业集聚区内村庄的农业人口成建制转为城镇户口；推行居住证管理制度，稳步推进城镇基本公共服务对常住人口全覆盖；建立健全农业人口转移促进机制，增强农民向城镇转移的动力。优化城市形态布局。坚持中心城市带动战略，统筹推进郑东新区开发建设，打造全省城乡一体化发展先行区；加快高新区、经开区等重点区域发展，提高产城融合水平；加快郑上新区规划建设，推动全市均衡发展；加快城市组团建设，各新城组团完成3至5平方公里起步区建设；做好县城中央商务区和十字景观大道建设，扎实推进“两环三十一放射”道路、生态廊道建设；实施老厂区、老居民区等城市重点片区改造，促进城市有机更新；探索各具特色的社区建设模式，在保护好历史文化风貌特色村前提下，启动项目70个、竣工40个；加快棚户区改造，建设安置房19.45万套。强化综合交通枢纽功能。加快构建“米”字形快速铁路网，配合建设郑徐高铁及郑开、郑焦、郑州至机场等城际铁路；加快推进国道107线郑州境东移、机场高速改扩建等干线公路和高速公路建设；加快“畅通郑州”工程建设，力争陇海路高架大学路以西段、京广快速化二期主体完工，开工建设金水路、花园路准快速和农业路快速路；全面实施中心城区188条支线路网打通工程，以地下管网和空间建设为重点升级改造一批城市道路，推进轨道交通5号线、2号线二期、1号线二期工程，争取3号线、南四环至郑州南站城郊铁路(2号线南延)早开工；新购新能源公交车500台，新增停车泊位3万个。提升城市综合承载能力。建成刘湾水厂、航空港第一水厂等项目，开工建设侯寨10万吨水厂、常庄20万吨水厂和邙山干渠复线工程等供水工程，争取南三环、马头岗二期等4个污水处理厂建成投用，推进坚强智能电网规划建设，开工建设北郊热源厂。提升城市管理水平。进一步发挥数字化城市管理平台作用，深入实施城市精细化管理；开展城市环境综合整治提升工程，确保机械化清扫率达到60%以上；强化城乡结合部、高速公路、铁路沿线、老旧小区、无主管楼院等环境综合整治。

　　(四)全面深化改革，着力增强发展内生动力。深化行政审批制度改革。持续简政放权，6月底前完成行政审批“两集中、两到位”改革；推动工商注册制度便利化，削减资质认定项目，把注册资本实缴登记制改为认缴登记制；建立市场主体准入负面清单制度，实行统一的市场准入；进一步放开公共服务市场准入，完善政府购买公共服务制度；在航空港实验区和经开区，探索试行“准入前国民待遇”和“负面清单”外资管理方式，放宽对外来投资的限制。深化土地管理、土地金融制度改革。深化城乡规划土地建设管理“六统一”改革，全面实施国有建设用地使用权网上交易招拍挂；力争年底前完成农村宅基地确权登记和颁证工作；开展农村集体土地进入市场流转试点工作，加快推进农村集体土地房屋登记；探索推行租让结合、分阶段出让的工业用地供应制度；建立健全国有土地资产经营制度和土地融资制度，探索土地使用权和承包经营权抵押融资制度。加快市属国有企业改革。深化市属国有企业公司股份制改革，完善国有资产管理体制，改革国有资本授权经营体制，探索国有企业重大信息公开，出台深化市管企业收入分配改革的指导意见。推进财税体制改革。实施全面规范、公开透明的预算制度，探索建立跨年度预算平衡机制，清理规范重点支出同财政收支增幅或生产总值挂钩事项，“三公”经费支出只减不增，继续深化国库管理制度改革，加强专项资金整合，全面落实“营改增”、资源税从价计征等改革政策。创新资金保障机制。鼓励具备条件的民间资本依法发起设立中小型银行等金融机构，研究建立城市基础设施、住宅政策性金融机构，做好永续债券、债贷结合等试点工作，推动设立中小微企业创新融资服务平台，力争全市小微企业贷款余额增速和全省占比均不低于35%。推进政府要素平台建设和资源性产品价格改革。做大做强郑州商品交易所，适时启动公共资源交易、产(股)权交易所等平台建设，稳妥推进城市供水、车用燃气、城市供热等资源性产品价格改革。全面深化农村综合改革。探索建立农村综合产权流转交易中心，创新农业投融资机制；鼓励农民专业合作社、农产品产地市场开展直供直销和电子商务等新模式；探索建立农村公共基础设施监管和“三资”管理运营新机制；继续深化集体林权制度改革，探索建立重点生态公益林管理和生态补偿机制。

　　(五)深入实施开放创新双驱动战略，着力提高对外开放水平和自主创新能力。全面实施开放驱动战略。加快开放平台建设，提升新郑综合保税区、出口加工区B区功能，完善提升铁路、航空一类口岸和综保区政策功能，积极申建经开综保区，以电子口岸为依托做好自贸区申报的前期准备工作，争取设立领事馆区和海外高层人才(专家)服务中心；拓宽开放领域，创新加工贸易模式，建设沿海加工贸易链条承接地；实施产业集群招商，围绕主导产业，瞄准国内外500强和行业前20强企业，聚焦战略支撑产业和新兴产业，大力引进行业龙头企业和配套企业，补链强链，提升业态，增强产业集群竞争力，力争在郑设立项目或机构的世界500强企业达到100家、谋划推动“三力”型项目100个、全年招商引资总规模突破1800亿元；提高经济外向度，优化进出口结构，培育竞争力强的外向型骨干企业，制定鼓励高附加值产品、服务产品和农产品出口政策，提高投资贸易便利化程度。大力实施创新驱动战略。进一步完善自主创新体系，加快建设国家重要的创新创业中心和创新型城市，争创国家促进科技和金融结合试点城市，科技进步对经济增长的贡献率达到59%；实施创新主体培育计划，重点培育1000家科技型企业，新建市级以上研发中心200家，力争在先进装备制造、电子信息等关键技术领域，取得一批具有自主知识产权的创新成果；大力发展高新技术产业，实施高新技术产业园区建设计划，积极培育新兴业态，争取高新技术产业年销售收入突破5600亿元；加快发展新型研发机构，支持组建一批产业技术创新联盟，建立一批产业技术创新研究机构；积极推进国家专利审协河南中心、国家技术转移郑州中心等创新载体平台建设；加快科技成果转化和产业化，建设一批科技公共服务机构，全年技术合同成交额达到110亿元；坚持人才强市战略，加大各类人才的引进和培育，实施创新人才引进计划，加大本土人才培育力度，引导激励科技人员创新创业。

　　(六)加强生态文明建设，着力改善生态环境。强化大气污染治理。加大燃煤锅炉拆改力度，年底完成市区燃煤锅炉拆改80台以上，减少市区用煤量28.8万吨，削减各类污染物排放量3168吨；加强黄标车治理，年底前淘汰全部公务黄标车，淘汰营运黄标车1000辆；强力推进扬尘治理，推广建筑工地扬尘污染防治技术，加强对渣土运输车辆监管；大力整治工业污染，加快电力、水泥等重点行业脱硫脱硝除尘改造，推动7家电力企业、15家水泥企业、4家水泥粉磨站实施脱硝和提标治理；加大对污染物排放不达标企业的执法力度，依照环保法规严格审批新建项目；深入推进“气化郑州”建设，大力推进节能减排项目，完成枣庄热源厂煤改气工程。扎实推进水生态文明建设。以建设全国水生态文明试点城市为契机，全面启动新一轮生态水系规划建设；加快花园口、赵口灌区等引黄、引水工程建设，发挥象湖、龙湖、圃田湖等平原水库调蓄作用，构筑郑州都市区生态水系循环利用系统；实施贾鲁河综合治理工程，全面完成潮河下游、枯河下游等河道综合治理，开工建设金水河、索须河、十八里河等重点区域水景观提升工程；加大截污治理力度，推动雨污水分流工程建设；全力推进南水北调配套工程建设，确保今年汛后与主体工程同步发挥作用。持续改善城乡环境。做好国家森林城市创建工作，力争今年通过考核验收；完成植树造林7.8万亩、森林抚育3.3万亩；续建树木花卉博览园、黄河国家湿地公园，完善提升10个森林公园，建设10个“走进森林”示范园；全面启动国家生态园林城市创建工作，建设一批以放射道路、铁路沿线、水系岸线为骨架的生态廊道、生态林带，新增绿地500万平方米；建成南环公园、西流湖公园，开工建设南水北调生态文化公园和27个区级综合性公园；编制生态农业建设规划，加大农业面源污染治理力度，启动农田资源智能监测系统建设；改善村庄人居环境，建设“美丽乡村”；严格生态环境源头保护，完善环境治理和生态修复制度。

　　(七)加快发展社会事业，着力保障和改善民生。继续加大财政投入，认真做好省、市“十大实事”等民生工作，力争全年民生投入占公共财政预算支出的比重达到70%以上。健全促进就业创业体制机制。加快推进公共就业和人才服务综合市场建设，突出做好高校毕业生就业和下岗职工再就业工作，重点抓好农村转移劳动力、城镇就业困难人员和退役军人就业工作，农村劳动力转移就业10万人，发放小额担保贷款10亿元。努力提高保障能力。实施以养老和医疗保险为重点的全民参保登记计划，扩大各项社会保险覆盖面，城乡低保、农村“五保”应保尽保；完善养老、优抚、助残、救孤、济困等制度，支持发展慈善事业；加强和改进价格调控监管，努力保持物价总水平基本稳定；完善住房保障制度，加大棚户区改造力度，加快以中小套型普通商品住房、公共租赁住房为主的住房建设，增强供给能力。优先发展教育。实施城乡基础教育一体化工程，新建、改扩建城乡幼儿园60所、市区中小学30所；推进义务教育质量综合评价改革，深入实施中小学课程改革；实施全民技能振兴和职业教育校企合作工程；做好郑州师范学院本科办学水平评估准备工作，推进旅游职业学院、职业技术学院新校区建设，扩建财税金融职业学院，做好中州大学升本和郑州卫校升专工作。提升健康水平。巩固新农合制度，持续推进新农合经办机制改革；丰富“片医”负责制社区卫生服务模式内涵，加强社区卫生服务中心建设；深入探索医疗集团建设，鼓励引导社会资本投资医疗服务市场，逐步组建1至3个区域医疗联合体；加强医疗基础设施建设，新增床位3000张。促进人口均衡发展。推进全国人口均衡型社会综合配套改革示范市建设，完善促进人口长期均衡发展政策，创新流动人口管理机制。促进文化繁荣。加快市民公共文化服务中心建设，推进公共文化服务体系提格升级；促进现代文化市场建设，提高文化产业创意能力和整体竞争力；组织文化精品创作生产，大力发展广播电视事业；办好嵩山论坛，建设华夏历史文明传承创新示范工程；扎实推进文化传承和“两园一中心”建设，做好大运河申遗工作。强化扶贫开发。全面实施贫困地区和革命老区扶贫规划，完成易地扶贫搬迁7293户26703人，安排整村推进20个，实施科技产业化扶贫项目45个，扶持新建贫困地区区域特色经济项目10个，帮助4.23万扶贫对象实现稳定脱贫。

　　(八)规范深化网格化管理，着力提升社会治理体系和治理能力现代化水平。以网格化管理、社会化服务为方向，强化社会公共管理信息平台应用，持续提升平台效能，促进政府职能在基层有效落实。加强城乡社区建设，强化基层自治服务功能。改革社会组织管理制度，激发社会组织活力。加强安全生产工作，严格落实领导分包制度，强化煤矿、非煤矿山、化工、消防等重点行业领域安全治理，坚决遏制重特大事故发生。强化食品药品安全监管，建立健全“黑名单”制度，实现全部药品和重要食品安全信息全过程可追溯。深入开展“六五”普法教育，做好法律援助工作。完善社会矛盾纠纷排查化解调处机制，健全信访工作制度，提高基层矛盾化解率。强力推进平安郑州建设，深化“四严一创”活动，维护社会和谐稳定。加强国防动员和国防后备力量建设，完成基干民兵调整改革任务，加大人民防空建设力度，推动军民融合深度发展。支持驻郑解放军、武警部队建设，深化“双拥”共建工作。持续推进民族团结进步创建活动，促进宗教和谐和睦。全面做好统计、审计、外事侨务、对台事务、社科研究、档案管理、防震减灾、机关事务管理和地方志等工作。

　　四、加强政府自身建设

　　认真贯彻落实党中央、国务院关于地方政府职能转变和机构改革的精神和要求，深入推进“两转两提”，全面提升城市治理能力和政府工作水平。

　　(一)转变政府职能，建设高效服务型政府。进一步理顺政府、市场、社会的关系，推动政府职能加快向创造良好发展环境、提供优质公共服务、维护社会公平正义转变。理顺职责关系，优化机构设置，全面完成政府机构改革任务。严控机构编制，确保财政供养人员只减不增。落实统一的市场准入制度，试行负面清单管理模式。进一步深化行政审批制度改革，加强政务服务体系建设，切实提高行政服务效能。

　　(二)推进依法行政，建设法治政府。坚持依法治市，提高依法行政水平。严格按照法定权限和程序行使权力、履行职责。严格执行人大及其常委会的决议决定，自觉接受法律和工作监督，主动接受人民政协民主监督，虚心接受社会监督，强化权力运行制约，完善政务公开制度。完善行政复议制度，深化行政复议试点工作。坚持民主立法、科学立法，提高政府立法质量。推行服务型行政执法，严格落实行政执法过错责任追究制。

　　(三)改进工作作风，建设为民务实清廉政府。扎实开展党的群众路线教育实践活动，严格执行中央八项规定和省委、市委贯彻意见，健全改进作风常态化制度，持续提升政府执行力和公信力。完善重大事项集体决策、专家咨询、社会公示等制度，提高政府决策民主化、科学化水平。加强廉政建设，完善惩防体系，强化行政问责、审计监督和效能监察，坚决查处权力寻租、权钱交易和损害群众利益的行为。坚持厉行节约，反对铺张浪费，严格控制“三公”经费支出。

　　(四)提高公务员队伍素质，建设学习创新型政府。深入创建学习型机关，强化教育培训，倡导务实重干，增强公务员队伍的宗旨意识、责任意识和履职能力。鼓励公务员到基层一线锻炼成长。加强和改进公务员管理，完善录用聘用、交流轮岗、竞争上岗等制度，健全创先争优长效机制，建设高素质公务员队伍。

　　各位代表！郑州的发展已经站在了一个新的战略起点。改革发展的美好前景鼓舞着我们，人民群众过上更加美好生活的热切期盼鞭策着我们。让我们紧密团结在以习近平同志为总书记的党中央周围，在省委、省政府和市委的坚强领导下，解放思想，振奋精神，锐意改革，奋发进取，为在全省率先全面建成小康社会、夺取郑州都市区建设新胜利而努力奋斗！
